# Auditory encoding abnormalities in children with autism spectrum disorder suggest delayed development of auditory cortex

**DOI:** 10.1186/s13229-015-0065-5

**Published:** 2015-12-30

**Authors:** J. Christopher Edgar, Charles L. Fisk IV, Jeffrey I. Berman, Darina Chudnovskaya, Song Liu, Juhi Pandey, John D. Herrington, Russell G. Port, Robert T. Schultz, Timothy P. L. Roberts

**Affiliations:** Lurie Family Foundations MEG Imaging Center, Department of Radiology, Children’s Hospital of Philadelphia, 34th and Civic Center Blvd, Wood Building, Suite 2115, Philadelphia, PA 19104 USA; Center for Autism Research, Department of Pediatrics, Children’s Hospital of Philadelphia, Philadelphia, PA USA

**Keywords:** Autism spectrum disorder, Development, Magnetoencephalography, Auditory

## Abstract

**Background:**

Findings of auditory abnormalities in children with autism spectrum disorder (ASD) include delayed superior temporal gyrus auditory responses, pre- and post-stimulus superior temporal gyrus (STG) auditory oscillatory abnormalities, and atypical hemispheric lateralization. These abnormalities are likely associated with abnormal brain maturation. To better understand changes in brain activity as a function of age, the present study investigated associations between age and STG auditory time-domain and time-frequency neural activity.

**Methods:**

While 306-channel magnetoencephalography (MEG) data were recorded, 500- and 1000-Hz tones of 300-ms duration were binaurally presented. Evaluable data were obtained from 63 typically developing children (TDC) (6 to 14 years old) and 52 children with ASD (6 to 14 years old). T1-weighted structural MRI was obtained, and a source model created using single dipoles anatomically constrained to each participant’s left and right STG. Using this source model, left and right 50-ms (M50), 100-ms (M100), and 200-ms (M200) time-domain and time-frequency measures (total power (TP) and inter-trial coherence (ITC)) were obtained.

**Results:**

Paired *t* tests showed a right STG M100 latency delay in ASD versus TDC (significant for right 500 Hz and marginally significant for right 1000 Hz). In the left and right STG, time-frequency analyses showed a greater pre- to post-stimulus increase in 4- to 16-Hz TP for both tones in ASD versus TDC after 150 ms. In the right STG, greater post-stimulus 4- to 16-Hz ITC for both tones was observed in TDC versus ASD after 200 ms. Analyses of age effects suggested M200 group differences that were due to a maturational delay in ASD, with left and right M200 decreasing with age in TDC but significantly less so in ASD. Additional evidence indicating delayed maturation of auditory cortex in ASD included atypical hemispheric functional asymmetries, including a right versus left M100 latency advantage in TDC but not ASD, and a stronger left than right M50 response in TDC but not ASD.

**Conclusions:**

Present findings indicated maturational abnormalities in the development of primary/secondary auditory areas in children with ASD. It is hypothesized that a longitudinal investigation of the maturation of auditory network activity will indicate delayed development of each component of the auditory processing system in ASD.

**Electronic supplementary material:**

The online version of this article (doi:10.1186/s13229-015-0065-5) contains supplementary material, which is available to authorized users.

## Background

Autism spectrum disorder (ASD) is a set of developmental disorders characterized by difficulties in social communication and social interaction and restricted, repetitive patterns of behavior, interests, or activities. Electroencephalography (EEG) and magnetoencephalography (MEG) studies consistently report auditory abnormalities in children with ASD. Findings include delayed superior temporal gyrus (STG) auditory responses [1–4], reduced STG 40-Hz auditory steady-state total power [5], pre- and post-stimulus pure tone STG oscillatory abnormalities [6], and atypical hemispheric lateralization of auditory responses [7]. To better understand the development of auditory abnormalities in ASD, the present study investigated associations between age and STG auditory processes. The following text reviews the auditory brain measures examined as well as findings regarding these auditory measures in ASD.

### Time-domain auditory measures

In adults, N100 (EEG) and M100 (MEG) are the most prominent deflections of the auditory event-related potential (EEG) or field (MEG), evolving with a peak latency of about 100 ms after stimulus onset (for a review, see [8, 9]). Naatanen and Picton [10] noted that although multiple brain regions contribute to the EEG N100, the major activity underlying the scalp-recorded N100 wave is located in the supratemporal plane. In adults, a smaller auditory response around 50 ms (EEG P1 or P50 and MEG M50) is often seen. The relevant MEG literature also points to STG as the primary P/M50 generator [11–16].

The maturation of primary/secondary auditory cortex involves a complex interplay of auditory event-related components. In young children, the P/M50 is readily evoked [17] and with the peak latency of this component in 5- and 6-year-old children at ~85–95 ms [18, 19]. P/M50 latency and amplitude decrease as a function of age [20, 21]. Although less common in young children, when present, the N/M100 response appears around 100–150 ms [2, 22, 23]. In older children, auditory responses become more complex and the components more defined, with an adult morphology typically observed around 10 to 12 years of age [22], and thus with a distinct N/M100 auditory response generally observed by late childhood and early adolescence [22, 24]. During this period of N/M100 maturation, the N/M200, a response occurring after N/M100 and with the same topography as N/M100, has a maximum amplitude at approximately 8 years and then decays until it is often not present in individuals 18 years and older [24, 25]. As detailed in the “Discussion” section, N/M200 is considered an endogenous response, associated with attention and cognition.

Delayed auditory responses have been observed in ASD. As an example, in an initial study from our laboratory, the right STG M100 auditory response was delayed by approximately 10 ms in children with ASD versus typically developing children (TDC) [3]. This latency delay was apparent for stimuli from 200 to 1000 Hz, although the largest group differences were observed for 300- and 500-Hz tones. Edgar et al. [6] examined the latency of M50 and M100 responses in 36 TDC and 105 children with ASD aged 6 to 15 years. In addition to replicating the right STG M100 latency delay finding, left and right STG M50 responses were also delayed by approximately 8 ms in ASD versus TDC. Despite these delays, in both groups, M50 and M100 showed a clear developmental trajectory. In particular, replicating previous findings [20, 22, 24], in Edgar et al., an association between the latency of the left and right STG M50 and M100 responses and age was observed in TDC and in ASD.

### Time-frequency auditory findings in ASD

Neural networks exhibit oscillatory activity over a wide range of frequencies: from delta-band activity (0 to 4 Hz) to at least gamma-band activity (approximately 30 to 50 Hz). Oscillatory activity observed in EEG/MEG recordings reflects synchronized neural activity that occurs over relatively short time periods (milliseconds). Oscillatory activity within specific frequency bands is one of the most promising candidate mechanisms associated with information processing (e.g., see [26, 27]) as well as brain dysfunction in clinical populations (e.g., see [28–30]). Several studies have reported auditory cortex oscillatory abnormalities in ASD. Presenting 40-Hz click trains to children with autism and age-matched controls aged 7 to 17 years, Wilson et al. [5] observed decreased left hemisphere 40-Hz steady-state gamma-band activity in ASD. Presenting 1000-Hz tones and examining the early STG transient gamma-band response, Rojas et al. [31] observed decreased left and right 40-Hz inter-trial coherence (ITC, also called phase-locking factor) in adults with ASD as well as in the parents of children with ASD, leading Rojas et al. to argue for a deficit in ASD in the timelocking of gamma oscillations to external stimuli. Replicating previous findings, in Edgar et al. [6], decreased post-stimulus superior temporal gyrus (STG) ~40-Hz evoked activity and ITC was observed bilaterally in children with ASD aged 6 to 16 years. Oscillatory abnormalities other than post-stimulus gamma abnormalities were observed in ASD including evoked and ITC low-frequency (below ~20 Hz) post-stimulus group differences from 100 to 200 ms. Greater STG pre-stimulus activity in ASD versus TDC was also observed (left and right hemisphere, 4–56 Hz), and increased pre-stimulus activity predicted a later M100 auditory response in both groups. It was hypothesized that greater pre-stimulus activity in ASD than TDC likely indicates a fundamental neuronal signal-to-noise deficit in individuals with ASD. Associations with age—greater pre-stimulus activity in younger than older ASD and TDC children—indicated a maturational component, and despite higher pre-stimulus 4- to 56-Hz activity in ASD, the age-related rate of change in pre-stimulus activity was similar in both groups.

### Study goals

The present study comprehensively investigated the maturation of auditory cortical responses in TDC and in children with ASD, examining auditory time-domain and time-frequency activity.

## Methods

### Participants

Of 126 examined participants, MEG data were excluded from five TDC and six children with ASD due to noise from dental metal artifacts (*N* = 6), no M50 and M100 response in either the left or right hemisphere (*N* = 1), and no structural magnetic resonance imaging (MRI) (*N* = 4). Evaluable MEG data were obtained from 63 TDC children (6 to 14 years old; 58 M/5 F) and 52 children with ASD (6 to 14 years old; 46 M/6 F). Demographics are reported in Table 1 for the evaluable sample.

**Table 1:** Demographic information

	TD (*N* = 63)		ASD (*N* = 52)			
	Mean	SD	Mean	SD	*t* value	*p* value
Age (years)	9.8	1.8	10.1	1.7	0.78	>0.05
SRS	40.8	6.2	75.8	11.7	20.51	<0.01
DAS-II GCA	112.6	14.3	107.0	22.3	1.62	>0.05

All participants were selected according to the following criteria: (1) no history of traumatic brain injury or other significant medical or neurological abnormality, (2) no active psychosis, (3) no MRI contraindications, and (4) no known drug or alcohol use prior to any study procedure. Members of the TDC group were evaluated by licensed clinical psychologists who ruled out the presence of DSM-IV-TR Axis I disorders based on clinical judgment, review of the child’s medical history form, and parent interview. Current diagnosis of ASD was confirmed by expert clinical judgment based on NIH CPEA guidelines, including the Autism Diagnostic Observation Schedule—Generic [32] and Autism Diagnostic Interview—Revised [33] and with consensus diagnostic agreement between at least two experienced clinicians. Additional details on participant recruitment procedures are provided in Edgar et al. [34].

In the ASD group, 17 participants were being treated with ADHD medications (e.g., Concerta, Strattera), 3 participants treated with second-generation antipsychotics (e.g., Risperidone), 12 participants with antidepressants (e.g., Prozac, Sertaline), and 1 participant with anxiolytics (Buspirone). No participants in the TDC group were taking psychotropic medications. The study was approved by the Children’s Hospital of Philadelphia IRB, and all participants’ families gave written consent.

### MEG and MRI data acquisition

MEG data were recorded using a 306-channel Vector View system (Elekta-Neuromag, Helsinki, Finland) with a sampling rate of 1000 Hz and a band-pass filter of 0.1 to 330 Hz. Electrooculogram (EOG) (vertical EOG on the upper and lower left sides) and electrocardiogram (ECG) were also obtained. The participants’ head position was monitored using four head position indicator (HPI) coils attached to the scalp.

Stimuli consisted of 500- and 1000-Hz sinusoidal tones presented using Eprime v1.1. Tones were presented via a sound pressure transducer and sound conduction tubing to the participant’s peripheral auditory canal via ear tip inserts (ER3A, Etymotic Research, IL, USA). Prior to data acquisition, 1000-Hz tones of 300-ms duration and 10-ms rise time were presented binaurally and incrementally until reaching auditory threshold for each ear. Tones were presented at 45-dB sensation level above threshold. Each trial consisted of a 500- or 1000-Hz tone (300-ms duration) plus a 1000-ms (±100) inter-trial interval. A total of 125 tones per condition were presented. The 500- and 1000-Hz tones were separately analyzed. To minimize fatigue, during the auditory test, participants viewed (but did not listen to) a movie projected onto a screen.

Following the MEG task, MEG data were corrected for head motion using MaxMove, and Maxfilter was used for noise reduction using a signal space separation method with a temporal extension (tSSS [35]). After motion correction and tSSS, artifact correction was applied to remove eye blink activity as outlined in Edgar et al. [6] using BESA. Non-eye blink artifacts were rejected by amplitude and gradient criteria (amplitude >1200 fT/cm, gradients >800 fT/cm/sample). Artifact-free epochs were then averaged, with MEG data analyzed only from participants with 50+ artifact-free trials. The number of artifact-free trials did not differ between TDC (500 Hz, mean = 116 (9.3); 1000 Hz, mean = 117 (9.3)) and ASD (500 Hz, mean = 117 (9.2); 1000 Hz, mean = 117 (9.9)), *p*s > 0.05.

After the MEG session, structural magnetic resonance imaging (sMRI) provided T1-weighted, 3D MPRAGE anatomical images for source localization (3T Siemens Verio scanner; voxel size 0.8 × 0.8 × 0.9 mm^3^).

### Source localization

Source localization was accomplished using anatomical constraints. To coregister MEG and sMRI data, three anatomical landmarks (nasion and right and left preauriculars) as well as an additional 200+ points on the scalp and face were digitized for each participant using the probe position identification (PPI) system (Polhemus, Colchester, VT), and a transformation matrix that involved rotation and translation between the MEG and sMRI coordinate systems was obtained via a least-squares match of the PPI points to the surface of the scalp and face.

For all participants, measures were obtained for the left and right 50-ms (M50), 100-ms (M100), and 200-ms (M200) response. The primary generator of the M50, M100, and M200 is well-modeled by a single dipole in the left and right Heschl’s gyrus and surrounding regions [18, 24, 36–38]. Therefore, after coregistering the MEG and sMRI data, each participant’s left and right Heschl’s gyrus was visually identified and a dipole source was placed at the “center” of Heschl’s gyrus at an anterior to posterior midpoint and approximately two thirds from the medial termination of Heschl’s gyrus. If two Heschl’s gyri were present, the dipole was placed between the two Heschl’s gyri. After placing the left and right dipoles, for each participant, left and right STG dipoles were oriented at the maximum of the M50, M100, and M200 response. Thus, although estimates of left and right STG activity were obtained using an anatomical constraint, orientation of the M50, M100, and M200 dipoles was optimized individually for each participant.

Dipole orientations were obtained after applying a 2 (24 dB/octave, zero phase) to 55-Hz (48 dB/octave, zero phase) band-pass filter. The presence of a M50 (35 to 125 ms) and M100 (80 to 195 ms) response was determined based on amplitude (greater than baseline), latency, and hemisphere ingoing and outgoing flux topography (e.g., for the M100 left hemisphere, ingoing anterior and outgoing posterior and vice versa for the right hemisphere). These slightly extended M50 and M100 latency ranges allowed capturing responses in younger children. M200 was operationally defined as the response showing a magnetic field topography similar to M100 but occurring after M100. As reported below, in many participants, a M100 response was not observed. In such instances, the M200 was defined as a response occurring later than 200 ms and with the appropriate magnetic field topography. Of note, as detailed in Edgar et al. [4], in some children, it is difficult to identify the M100 response with 100 % certainty, especially in younger subjects where M100 just emerges from M200. In the present study, M100 was scored as present if there was a peak with a rising and falling slope distinct from the M200, with a M100 magnetic field topography and with a latency between 80 and 195 ms. In the present study, in the few cases of ambiguous M100 determination, the final score was determined by consensus review.

When a M50 or M100 response was observed, left and right M50 (35–125 ms) and M100 (80–195 ms) amplitude was measured at the signal maxima and latency the time at which this maximum occurred, within each latency interval. Examination of M200 source waveforms indicated that for most participants, the M200 response occurred between 200 and 325 ms and that for many participants, the M200 response was broad (i.e., extended over a long period) and often not very “peaked”. Due to this feature, M200 latency was not scored. For M200 amplitude, for each participant, in the 200- to 325-ms window, a source strength measure integrated over the full-width at half-max (FWHM) of the M200 response rather than selecting a single timepoint amplitude measure. For left and right STG M50, M100, and M200, the amplitude measure was obtained after baseline correction (−400 to −25 ms).

### Source time-frequency analysis

The calculation of single-trial phase and magnitude for the left and right sources used procedures outlined in Hoechstetter et al. [39] where in each participant, the derived source model was applied to the raw unfiltered data. Each source model was constructed by including left and right Heschl’s gyrus sources and the eye blink source vector derived for each participant to remove eye blink activity [40, 41]. This final source model served as a source montage for the raw MEG [42, 43]. Examining the source waveforms, transformation from the time-domain to the time-frequency domain used complex demodulation procedures [44] implemented in BESA, using frequencies between 4 and 56 Hz, in steps of 2 Hz. Continuous data were analyzed relative to tone onset every 25 ms (i.e., each 40-Hz cycle), utilizing ±39.4 ms and ±2.83 Hz (full-width at half-maximum parameters) of contiguous data at each 25-ms step.

Total power and phase-locking measures were extracted from the single-trial complex time-frequency matrix. Total power was calculated by averaging the time-frequency spectra of each MEG epoch. When baseline power is subtracted, post-stimulus total power (TP) assesses the post-stimulus increase in oscillatory activity. A measure of phase-locking referred to as ITC was also computed. ITC is a normalized measure assessing the trial-to-trial similarity of oscillatory activity, with ITC = 1 reflecting no phase variability and ITC = 0 reflecting maximal phase variability across trials. Time-frequency measures were obtained using the M50, M100, and M200 source models.

For the TP and ITC time-frequency *t* test group comparisons, family-wise error was controlled using cluster size thresholds derived from Monte Carlo simulations (e.g., see [6, 28]). The method computes the probability of a random field of noise producing a cluster of adjacent time-frequency cells of a given size after the noise is thresholded at a given probability level and provides a corrected *p* value. The cluster size needed to obtain the desired family-wise correction was determined using a standard functional magnetic resonance imaging (fMRI) package (AFNI AlphaSim) and clustering performed with custom MatLab software.

For all following analyses, participants with values more than 2.5 standard deviations were excluded (typically one to two participants per analysis).

## Results

### Demographics

As shown in Table 1, groups did not differ in age or IQ (DAS-II General Cognitive Ability). As expected, Social Responsiveness Scale scores (SRS [45]) were significantly higher in ASD than those in TDC.

### Source localization

For the 500- and 1000-Hz tones, bilateral M50 and M100 goodness-of-fit (GOF; evaluated over all sensor locations) did not differ between ASD and TDC (ASD range 63 to 76 %; TDC range 65 to 74 %; *p*s >0.05). For the 500- and 1000-Hz tones, M200 GOF was slightly lower in ASD (500 Hz = 76 % and 1000 Hz = 74 %) versus TDC (500 Hz = 80 % and 1000 Hz = 81 %; *p*s <0.05). As described below, the slightly lower M200 GOF in ASD was likely due to more extended and less synchronous M200 responses in ASD versus TDC.

### M50 and M100: present versus absent responses

As shown in Table 2 upper panel, whereas M50 responses were observed in almost all participants, M100 responses were observed much less often. McNemar tests indicated that 500- and 1000-Hz M100 responses were present more often in the right than in the left STG (*p*s <0.001). As shown in Table 2 lower panel, the presence versus absence of a M100 response was associated with age, with a median split by age indicating 500- and 1000-Hz responses more often in older (>10 years old) than younger participants (*p*s <0.001). Analyses indicated similar age dependence of the presence of a M100 response in TDC versus ASD. The presence of a M200 response is not reported in Table 2 as a M200 was observed in all but one participant.

**Table 2:** Presence of M50 and M100

	500 Hz		1000 Hz	
	Left STG (%)	Right STG (%)	Left STG (%)	Right STG (%)
M50	96	94	96	95
M100	39	62	45	68
M100 younger	27	48	33	65
M100 older	53	77	60	72

### Time-domain findings: M50, M100, M200 latency and amplitude

#### Latency

ANOVAs examined hemisphere, frequency, and group latency differences in the participants with M50, M100, or M200 responses.

##### M50

A main effect of hemisphere, *F*(1,99) = 16.49, *p* < 0.001, showed earlier M50 responses in the right versus left STG. A main effect of frequency, *F*(1,99) = 67.01, *p* < 0.001, showed earlier M50 responses for 1000-Hz versus 500-Hz tones.

##### M100

A trending group × hemisphere interaction, *F*(1,32) = 3.02, *p* = 0.09, showed earlier right than left response latencies in TDC (*p* < 0.001) and similar right and left response latencies in ASD (*p* > 0.05).

Table 3 shows M50 and M100 mean and standard deviation latency values for each group. As shown in Table 3, paired *t* tests showed the expected right hemisphere M100 latency delay in ASD versus TDC (significant for right 500 Hz and marginally significant for right 1000 Hz).

**Table 3:** Latency: M50 and M100

	*N*	M50 500 Hz	*N*	M50 1000 Hz	*N*	M100 500 Hz	*N*	M100 1000 Hz
	M50	Latency(ms) and SD	M50	Latency (ms) and SD	M100	Latency (ms) and SD	M100	Latency (ms) and SD
Controls								
Left STG	57	93 (15)	60	87 (16)	25	151 (29)	33	141 (27)
Right STG	57	88 (12)	60	80 (13)	38	124 (14)*	45	120 (17)
ASD								
Left STG	45	93 (18)	47	85 (17)	22	144 (30)	27	131 (32)
Right STG	46	89 (15)	48	80 (14)	34	132 (19)*	33	127 (23)

*Comparing TDC to ASD, significant right hemisphere M100 500-Hz group latency differences were observed (*p* = 0.05)

#### Amplitude

ANOVAs examined hemisphere, frequency, and group amplitude differences in the participants with M50, M100, or M200 responses.

##### M50

A group × hemisphere interaction, *F*(1,99) = 6.23, *p* < 0.01, indicated stronger left than right M50 responses in TDC but not ASD (*p* < 0.01). A hemisphere × frequency interaction, *F*(1,99) = 5.34, *p* < 0.05, indicated stronger 1000- than 500-Hz responses in the right (*p* < 0.001) and similarly strong 1000- and 500-Hz responses in the left (*p* > 0.05).

##### M100

A main effect of frequency, *F*(1,29) = 4.82, *p* < 0.05, indicated stronger M100 responses to 1000- than 500-Hz tones.

##### M200 (FWHM)

**Fig. 1:**
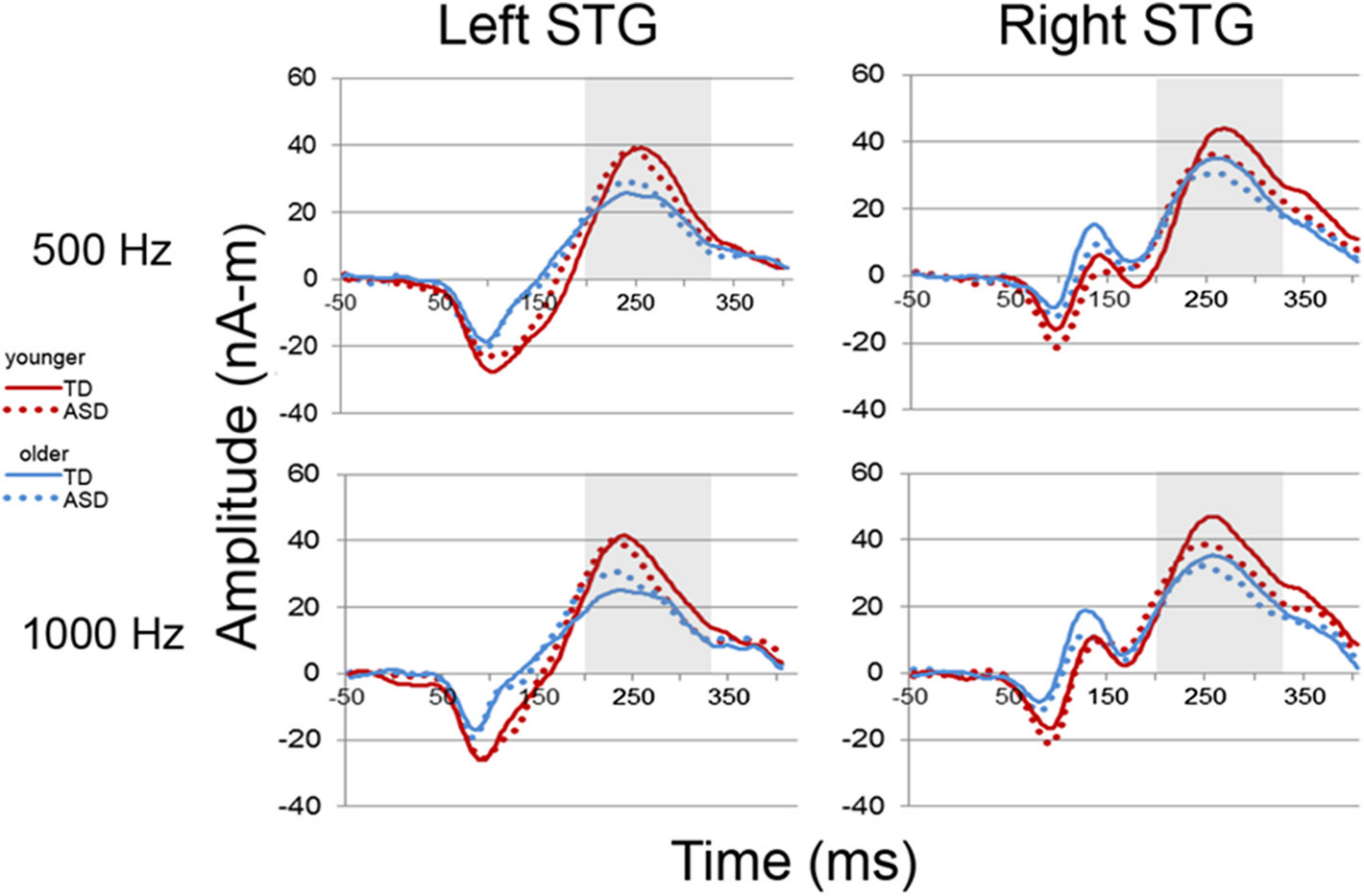
Left and right M200 source waveforms shown for the 500- and 1000-Hz tones, for older (>10 years old, *blue*) and younger participants (*red*) and for TDC (*solid line*) and ASD (*dotted line*). Time (ms) is shown on the *x*-axis and source strength (nA-m) on the *y*-axis. The M200 interval (200 to 325 ms) is highlighted. Repeated measures ANOVA showed a trending main effect of amplitude with stronger M200 responses in TDC than ASD. Of note, mirroring the findings reported in Table 1, grand average waveforms show a M100 response in the right but not left hemisphere and more prominent in older than younger participants

A main effect of hemisphere, *F*(1,108) = 30.12, *p* < 0.001, indicated stronger M200 responses in the right versus left. A trending main effect of group, *F*(1,108) = 2.68, *p* = 0.10, indicated stronger M200 responses in TDC versus ASD. Figure 1 shows M200 grand average left and right source waveforms for each group and each tone.

### Brain function time-frequency analyses

**Fig. 2:**
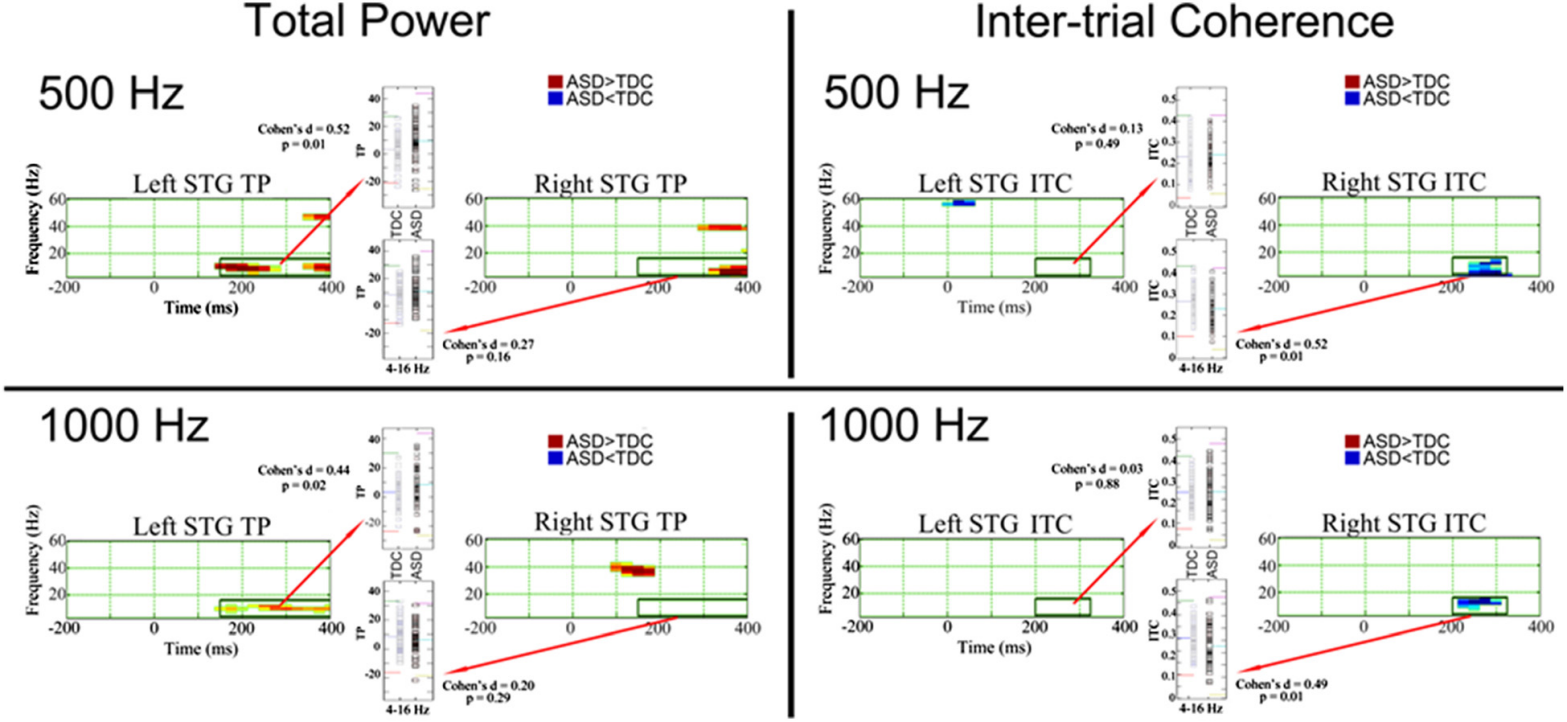
The *left panel* shows TP family-wise corrected statistical maps for left and right STG and for 500- and 1000-Hz tones (ASD > TDC red). Time is shown on the *x*-axis and frequency on the *y*-axis. *Insets* show STG post-stimulus low-frequency TP values for each participant (4- to 16-Hz activity averaged from 150 to 400 ms). In the inset, *colored lines* show the mean and ±2 SD. The *right panel* shows inter-trial coherence family-wise corrected statistical maps. *Right panel insets* show STG post-stimulus low-frequency inter-trial coherence values for each participant (4- to 16-Hz activity averaged from 200 to 325 ms)

Given absent M100 responses in many participants, time-frequency analyses were not performed for M100 sources. Given M200 amplitude group differences bilaterally, to reduce the number of tests, time-frequency analyses were performed only for M200 sources (as shown in Additional file 1: Figure S1, the axis of M50 and M200 dipole orientations were very similar (dipoles are oriented at 180° to each other), indicating that M50 and M200 sources would provide very similar estimates of STG activity). Figure 2 left panel shows family-wise error-corrected M200 TP statistics maps (ASD > TDC red). The left and right TP statistics maps show a greater pre- to post-stimulus increase in 4- to 16-Hz activity in ASD than TDC after 150 ms (except for right STG 1000 Hz). Insets show left and right post-stimulus low-frequency TP values for each participant (4- to 16-Hz activity averaged from 150 to 400 ms). Figure 2 right panel shows family-wise corrected M200 ITC statistics maps. Reduced right post-stimulus 4- to 16-Hz ITC was observed in ASD versus TDC after 200 ms for both tones (ASD < TDC blue). Insets show left and right post-stimulus low-frequency ITC values for each participant (4- to 16-Hz activity averaged from 200 to 300 ms). (Additional file 2: Figure S2 shows TP and ITC images for each group and hemisphere.) In addition to the above, Fig. 2 left STG 500 Hz family-wise corrected statistics plot shows reduced post-stimulus transient gamma ITC from ~25 to 100 ms in ASD versus TDC.

To further explore the post-stimulus low-frequency group differences, ANOVAs examined hemisphere, condition (500 Hz, 1000 Hz), and group low-frequency differences using a single measure per hemisphere identified from the family-wise corrected statistics maps, computed for TP as the average in a 150- to 400-ms and 4- to 16-Hz region of interest (ROI), and computed for ITC as the average in a 200- to 300-ms and 4- to 16-Hz ROI. For TP, a main effect of condition, *F*(1,110) = 3.81, *p* < 0.05, indicated greater TP for the 500- versus 1000-Hz tones. Simple effect analyses of a hemisphere by group interaction, *F*(1,110) = 7.60, *p* < 0.01, showed greater left STG TP in ASD versus TDC (*p* < 0.05) and greater right than left TP in TDC (*p* < 0.01) but not ASD (*p* > 0.05). For ITC, simple effect analyses of a hemisphere by group interaction, *F*(1,112) = 15.43, *p* < 0.001, showed greater right STG ITC in TDC versus ASD (*p* < 0.05) as well as greater right than left ITC in TDC (*p* < 0.001) but not ASD (*p* > 0.05). Given no group × condition interactions for TP or ITC, to reduce the number of tests, all further analyses were performed collapsing across 500- and 1000-Hz conditions. Table 4 shows ANOVA results for the averaged TP and ITC measures.

**Table 4:** Group differences and associations with age

	Group and hemisphere differences								
	Group			Hemisphere			Group × Hemisphere		
	*F*	*p* value	Effect	*F*	*p* value	Effect	*F*	*p* value	Effect
Post-stimulus TP	2.54	0.11	NA	3.66	0.06	See interaction	10.04	<0.01	Left (ASD > TDC)
Post-stimulus ITC	0.45	0.51	NA	4.85	0.03	See interaction	15.43	<0.001	Right (ASD < TDC)
Pre-stimulus activity	0.38	0.54	NA	9.76	<0.01	(left > right)	0.03	0.86	NA
	Associations with age								
		Age		Group			Age × Group		
	Hemisphere	*r*	*p* value	*r* ^2^ change	*p* value	Effect	*r* ^2^ change	*p* value	Effect
Post-stimulus TP	L	0.3	0.001	0.05	0.02	ASD > TDC	0.02	0.17	NA
	R	0.14	0.13	0	0.9	NA	0.01	0.28	NA
Post-stimulus ITC	L	0.38	<0.001	0.02	0.16	NA	0.01	0.36	NA
	R	0.22	0.02	0.03	0.05	TDC > ASD	0.03	0.04	TDC r = −0.43, ASD *r* = 0.00
Pre-stimulus Activity	L	0.07	0.5	0	0.61	NA	0.01	0.27	NA
	R	0.2	0.04	0	0.73	NA	0	0.77	NA

Given the pre-stimulus TDC and ASD group findings reported in Edgar et al. [6], a pre-stimulus measure (4 to 56 Hz) was also examined, computed by time-frequency transforming each trial and then averaging (i.e., a pre-stimulus total power measure). Given that the 500- and 1000-Hz tones were randomly presented, a single pre-stimulus measure was obtained by averaging across the 500- and 1000-Hz conditions. As shown in Table 4, a main effect of hemisphere indicated greater left than right pre-stimulus activity. No pre-stimulus group differences were observed.

Given previous findings that pre-stimulus activity predicts post-stimulus STG auditory processes [6], regressions examined associations between pre- and post-stimulus activity (pre-stimulus activity first block, group second block, interaction term third block, post-stimulus TP and ITC measures the dependent variable). In the left, a significant first block main effect, *F*(1,109) = 50.16, *p* < 0.01, indicated that less pre-stimulus activity was associated with increased post-stimulus TP in both groups (*r* = 0.21). In the left, a significant second block main effect, *F*(1,108) = 3.87, *p* < 0.05, indicated that left TP post-stimulus group differences remained even after removing variance associated with pre-stimulus activity. In the left, a significant first block main effect, *F*(1,109) = 12.29, *p* < 0.001, indicated that less pre-stimulus activity was associated with increased ITC in both groups (*r* = 0.32). The left STG findings remained even after removing variance in post-stimulus activity associated with age. Age + pre-stimulus activity together explained 13 and 25 % of the variance in left TP and ITC, respectively. Right pre-stimulus activity was not associated with right TP or ITC. A significant second block main effect, *F*(1,108) = 4.05, *p* < 0.05, indicated that right ITC post-stimulus group differences remained even after removing variance associated with pre-stimulus activity.

### Brain function: associations with age

**Fig. 3:**
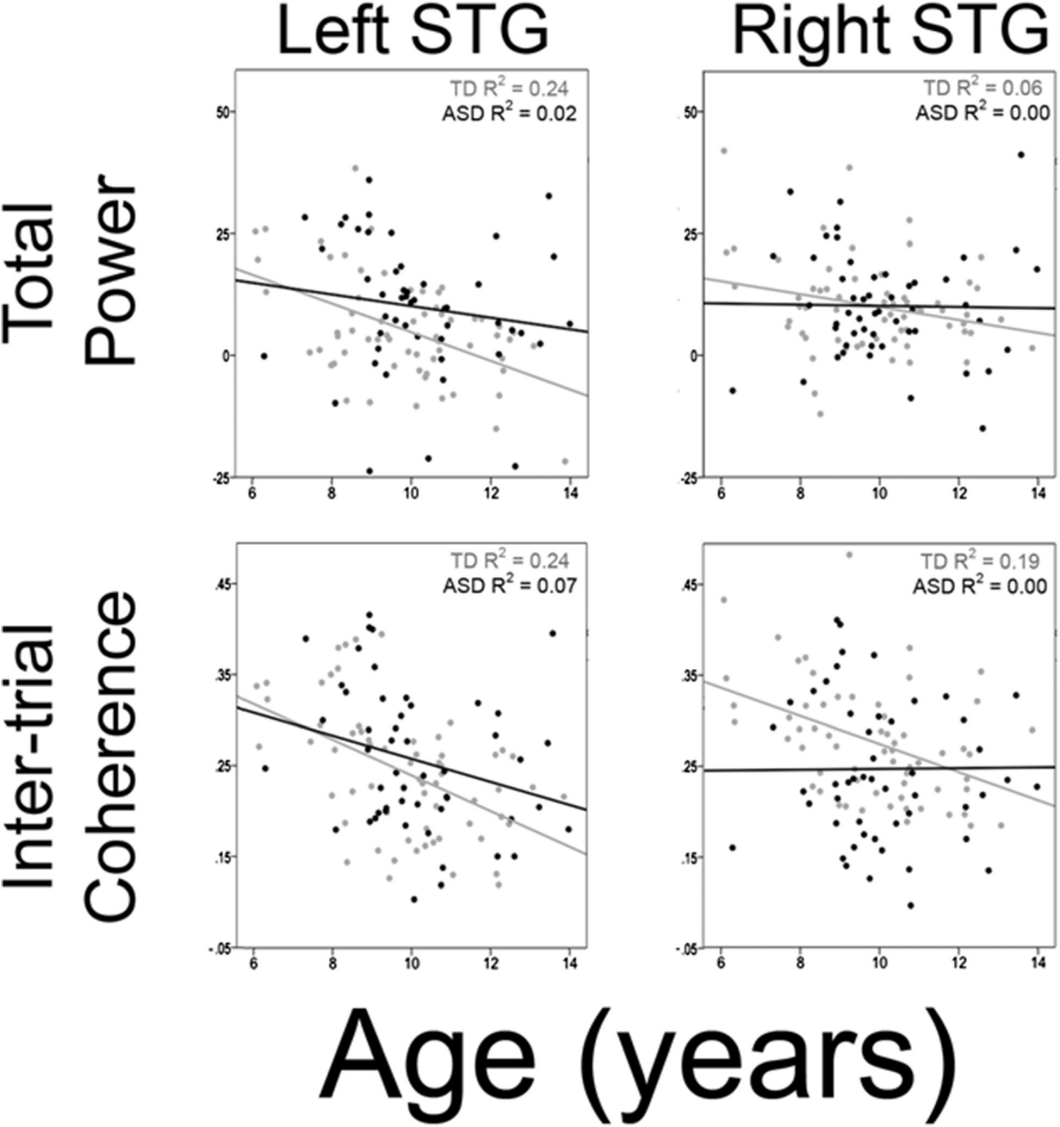
Scatterplots showing associations between age and left and right M200 TP (*upper row*; 4- to 16-Hz activity averaged from 150 to 400 ms; age on *x*-axis and TP on *y*-axis) and M200 ITC (*bottom row*; 4- to 16-Hz activity averaged from 200 to 300 ms; age on *x*-axis and ITC on *y*-axis). Associations are shown for TDC (*light gray*) and ASD (black)

Figure 3 scatterplots show TP and age (top row) and ITC and age (bottom row) associations for each group (Additional file 3: Figure S3 and Additional file 4: Figure S4 show associations with age for time-domain M50, M100, and M200 latency and amplitude; Additional file 5: Figure S5 shows scatterplots for each 500- and 1000-Hz condition). To examine associations with age, for each hemisphere, regressions were performed with age entered first, group second, the age × group interaction term last, and with the average M200 TP or ITC ROI value as the dependent measure. As shown in Table 4, TP and age associations were only observed for left TP (although the Fig. 3 scatterplot suggests an association in TDC (*r*^2^ = 0.24) versus ASD (*r*^2^ = 0.02), the interaction term was not significant, and as detailed in the “Discussion” section and Additional file 6, this was likely due to slightly insufficient statistical power). ITC and age associations were observed for TDC and ASD in the left and for only TDC in the right. Table 4 “Group” column shows that the left TP and the right ITC group differences remained after removing variance in TP or ITC associated with age. Finally, Table 4 shows that increased age was associated with decreased right pre-stimulus activity.

An additional set of analyses examined associations between post-stimulus TP and ITC (using the above defined M200 time-frequency ROIs). Given the above reported age, pre- and post-stimulus activity associations, for each hemisphere, a regression model examined whether post-stimulus TP predicted variance in post-stimulus ITC after removing variance associated with pre-stimulus activity and age (pre-stimulus activity and age entered first block, post-stimulus TP second block, and the group × post-stimulus TP interaction entered third block). Post-stimulus TP predicted unique variance in left (23 %) and right (44 %) post-stimulus ITC. This positive association likely reflects the fact that (1) post-stimulus TP is computed as a percent change from pre-stimulus activity, with less pre-stimulus activity “allowing” a greater pre- to post-stimulus change in power and (2) phase is more accurately estimated a higher versus lower signal-to-noise and thus ITC more accurately estimated in those individuals with stronger versus weaker post-stimulus activity [46]. Non-significant interactions (*p*s >0.23) indicated that increased post-stimulus TP was associated with increased post-stimulus ITC in both groups.

## Discussion

The present study examined the maturation of STG auditory responses in ASD. Cross-sectional findings indicated an array of abnormalities in the development of primary/secondary auditory function in children with ASD, including delayed right STG M100 responses and a reduced rate of change (and perhaps even no change) in left and right STG M200 activity as a function of age. Additional evidence indicating delayed auditory cortex maturation in ASD included atypical hemispheric functional asymmetries, including a right versus left M100 latency advantage in TDC but not ASD, and a stronger left than right M50 response in TDC but not ASD. The pattern of findings suggests the need for longitudinal studies investigating the maturation of auditory network activity in ASD, with the hypothesis that results from these studies will demonstrate delayed development of each component of the auditory processing system.

In the present study, a novel, and perhaps the most robust finding, was abnormal maturation of the networks supporting low-frequency (4 to 16 Hz) M200 neural activity in ASD versus TDC. In particular, whereas in TDC, low-frequency M200 TP and ITC decreased as a function of age (resulting in a decreased M200 time-domain response over the course of development); these age-associated TP and ITC changes were less evident or were absent in ASD (see Fig. 3). There was also evidence that the primary contribution to abnormal M200 activity in ASD differed between the hemispheres, with left M200 group differences primarily driven by left M200 TP decreasing more slowly as a function of age in ASD versus TDC (Fig. 3, upper left panel), and with right M200 group differences primarily driven by right M200 ITC decreasing as a function of age in TDC but not in ASD (Fig. 3, lower right panel). Whether these trajectory differences relate to anomalous neural circuitry in ASD or simply to a delay in development remains to be elucidated.

EEG studies examining typically developing children show that N2 amplitude decreases as a function of age [23, 47, 48]. For example, Čeponienė et al. [18] reported that N2 amplitude decreases from age 4 years through adulthood and Enoki et al. [49] observed N2 responses only in subjects younger than 18 years. The reason for this decrease, with a N2/M200 response often not observed in adults, is unclear. Some researchers have hypothesized that this decline is related to a drop in synaptic density after 10 years of age [22] or to the development of frontal inhibitory control. The absence of age-related M200 decrease in ASD may relate to atypical (or diminished) inhibitory connectivity between frontal and temporal regions, with a frontal contribution to M200 hypothesized given evidence for associations between reduced N2 amplitude and better performance on attention tasks [50]. For example, Johnstone et al. [25] reported an association between increased age and smaller N2 responses in children aged 8 to 17 years old as well as an association between decreased N2 amplitude and decreased reaction time and decreased number of errors on a target detection task. Johnstone et al. hypothesized that an age-associated decrease in N2 reflects maturation of stimulus discrimination abilities (e.g., age-related increase in ability to control the direction of attention). In the present study, the weak or absent associations between M200 time-frequency measures and age may be associated with a maturational delay (or absence of maturation) in stimulus discrimination processes in ASD. Source localization studies obtaining behavioral measures in children with ASD during an auditory signal detection task are needed.

Also indicating delayed maturation of auditory cortex in ASD was the observation of an absence of hemisphere functional asymmetries that are typically observed in children. First, replicating a previous finding [1], the absence of hemisphere asymmetry was observed in right versus left M100 latency advantage in TDC but not ASD. Second, and again replicating a previous finding [51], a stronger left than right M50 response was observed in TDC but not ASD. Regarding the M50 laterality findings, M50 strength decreases as a function of age (with M50 often absent in adults), with a larger left than right M50 response in this and other studies examining typically developing children and adolescents indicating earlier development of right than left primary/secondary auditory cortices. Examination of auditory processing asymmetries in other neurodevelopmental groups (e.g., XYY, learning disorders, ADHD) is needed to determine if the loss of these functional left-right STG asymmetries are specific to ASD or if they instead reflect abnormal brain maturation processes common to neurodevelopmental disorders [52, 53].

Other auditory cortex hemisphere asymmetries often observed in typically developing children, however, were observed to be intact in ASD. As an example, a M100 response was absent in many TDC and ASD participants, with M100 responses more often present in the right than left hemisphere and with M100 responses more often present in older (>10 years old) than younger participants. The latter findings indicated that the older individuals more easily encoded the auditory stimuli, with present findings thus consistent with prior studies showing that 100-ms auditory generators require longer intervals to produce a response in children than adults given hypothesized longer refractory periods in children than adults [20, 54, 55]. Present M100 findings are also generally consistent with Sharma et al. [56], who observed a 100-ms response (Fz N1a) in approximately 61 % of 6–7-year-olds and 69 % of 10–12-year-olds, but with the present findings extending Sharma et al. to show hemisphere differences, with earlier development of right than left STG auditory areas.

In the present study, the presence/absence of M100 was similar between TDC and ASD. Edgar et al. [4], however, reported absent M100 responses more often in children with ASD with language/cognitive impairment versus TDC. Study differences likely reflect the fact that a much higher functioning group of children with ASD was examined in the present study (average IQ = 113) versus Edgar et al. [4] (average IQ <100). Of note, however, present results replicated findings of a later right M100 response in children and adolescents with ASD versus TDC [3, 6], more clearly observed for right 500- than 1000-Hz tones. This replication is of note, as the present study used a different MEG system (Elekta) than previous studies (CTF), and the present study estimated left and right auditory activity using anatomical constraints (i.e., dipole sources placed at each participant’s left and right Heschl’s gyrus) versus a standard source model used in previous studies.

Two other findings observed in both TDC and ASD suggested faster maturation of right than left STG auditory areas: earlier M50 responses in the right than left hemisphere and less right than left pre-stimulus activity. The left versus right STG M50 latency difference observed in the present study has been reported in previous studies examining children and adolescents [50, 51]. As an example, comparison of the source localized left and right STG P1 latencies in Tables 1 and 2 of Albrech et al. [57] shows an approximately 10 ms earlier right than left P1 response in children 5 to 10 years old, with similar left and right P1 STG latencies not observed until about 16 years old. Regarding pre-stimulus activity, the observation of greater left than right pre-stimulus activity as well as the observation of an age-related decrease in pre-stimulus STG activity in the present study replicates Edgar et al. [6]. Different from Edgar et al., however, is that in the present study, pre-stimulus activity was not significantly decreased in TDC versus ASD. A failure to observe pre-stimulus group differences in the present study is perhaps due to the use of axial gradiometers in Edgar et al. [6] versus planar gradiometers in the present study, with axial gradiometers having broader sensitivity, with study differences thus suggesting that axial gradiometers provide a more comprehensive assessment of pre-stimulus activity (brain noise). Similar to the pre-stimulus findings in Edgar et al. [6], however, in both groups, decreased pre-stimulus activity predicted post-stimulus responses more typical of older versus younger children. For example, in the left STG, increased pre-stimulus activity was associated with increased M200 ITC. It is hypothesized that as the brain matures, neural networks become more efficient, with increased network efficiency associated with decreased pre-stimulus activity and with increased network efficiency resulting in stronger and more synchronous post-stimulus activity. Later development of left auditory cortex activity—assessed in terms of pre-stimulus activity and post-stimulus latency and amplitude—may allow a longer period of auditory cortex maturation, thereby facilitating left-hemisphere language specialization.

Given changes in auditory cortex activity as a function of age, it is hypothesized that the pattern of TDC versus ASD group differences in auditory cortex activity (including group differences in hemisphere asymmetries) will differ as a function of the age, with some auditory encoding impairments most clearly observed in younger children with ASD (e.g., the M200 group differences observed in the present study), and other impairments more evident in older children with ASD. A review of the literature supports this hypothesis. For example, although in the present study, the observation of a delayed right M100 response in ASD versus TDC replicates previous studies [3, 4], the M100 response in the present study was absent in many of the younger subjects, indicating that M100 group differences are better evaluated in older participants. A recent longitudinal study provided support for this claim, where it was observed that M100 latency abnormalities in ASD were more clearly observed in older versus younger children [58]. As another example, regarding early post-stimulus time-frequency gamma activity, in the present study, there was weak evidence for greater post-stimulus (~25 to 150 ms) gamma ITC in TDC than ASD, with group differences observed only for the left 500-Hz tone. Some differences between the present findings versus other studies more clearly showing post-stimulus gamma abnormalities in ASD may be due to examining a younger population in the present study versus the older (including adult) populations examined in previous studies [5, 31]. Evidence in support of auditory post-stimulus gamma group differences more clearly observed in older versus younger groups is provided in Port et al. [58]. In addition, a study reporting on 40-Hz steady-state STG activity from a smaller subgroup of the present sample found very weak or no 40-Hz steady-state activity in the younger versus older TDC and ASD children, indicating that 40-Hz auditory driving tasks are not optimal for examining 40-Hz circuits in young children (Edgar et al., in press). Thus, given the above, it is likely that different auditory measures will best identify group membership at different ages.

### Limitations and future directions

A limitation of the present study is that analyses were cross-sectional rather than longitudinal. A longitudinal study examining changes in auditory processes as a function of age in TDC and in children with ASD 5 to ~12 years old would support and extend the present cross-sectional findings. Future studies examining auditory processes in infants and children also are of interest given that treatments seeking to normalize auditory processes in ASD will likely target younger rather than older populations in order to have a better chance at normalizing auditory processes. In these studies, use of a whole-head infant MEG system is preferred given that MEG sensors are closer to the head in infant versus adult MEG systems [59–63].

Although a movie was shown during the task to keep participants comfortable and awake (no sound, only subtitles), another study limitation is that group differences attending to the movie (not assessed) could account for some study findings, such as decreased attention to the movie and increased attention to the tones in one group contributing to the M200 group differences. Studies assessing group differences in attention during such tasks (perhaps using eye tracking) are needed.

Finally, sample size was a slight limitation. In particular, although samples were moderately large, the present study was slightly underpowered for some analyses, especially for analyses involving interaction terms. As an example, as previously noted, although examination of the Fig. 3 upper left panel indicates decreased M200 TP in TDC but not ASD, the age × group interaction term was not significant, likely due to insufficient power. Indeed, power analyses indicate that given the observed TDC and ASD correlations and a sample of 55 per group, the present study was only slightly underpowered to detect this left TP interaction (TDC *r* = 0.49, ASD *r* = 0.14, power = ~78 %; see also Additional file 6 for further details). Assuming similar group effects in future studies, power analyses indicate that for all of the trending findings, a sample of 60 per group would provide sufficient power (>0.80) in cross-sectional designs. Of course, power would also be improved using a longitudinal design.

## Conclusions

Present cross-sectional findings showed an array of abnormalities in the development of primary/secondary auditory areas in children with ASD. Longitudinal studies, examining left and right auditory encoding processes from infancy through late adolescence (and also assessing hemisphere differences in trajectory) are needed to fully understand auditory encoding abnormalities in ASD. It is hypothesized that investigation of the maturation of auditory network activity will indicate delayed development of each component of the auditory processing system in ASD.

## Additional files

Additional file 1: Figure S1. For both the M50 and M200, the azimuthal dipole angle $\left(\theta =\mathrm{t}\mathrm{a}{\mathrm{n}}^{-1}\left(\frac{z\kern0.5em \mathrm{component}}{y\kern0.5em \mathrm{component}}\right)\right)$ was examined as this best captures the variability in the tangentially oriented STG M50 and M200 dipole orientations. For each participant, the difference in the M50 and M200 dipole orientation was computed for the left and right hemisphere. The histogram shows very similar left and right M50 and M200 dipole orientations for almost all participants. (TIF 49 kb) Additional file 2: Figure S2. The left panel shows grand average TP plots and the right panel ITC plots for each tone and each group. (TIF 338 kb) Additional file 3: Figure S3. Scatterplots showing associations for each tone between age and left and right M50 latency (upper row) and M100 latency (center row). Associations are shown for TDC (light gray) and ASD (black). The *x*-axis shows age and the *y*-axis latency. (TIF 128 kb) Additional file 4: Figure S4. Scatterplots showing associations for each tone between age and left and right M50 amplitude (upper row), M100 amplitude (center row), and M200 amplitude (bottom row). Associations are shown for TDC (light gray) and ASD (black). The *x*-axis shows age and the *y*-axis amplitude. For M200, the source strength measure is integrated over the full-width at half-max. (TIF 150 kb) Additional file 5: Figure S5. Upper row scatterplots show associations for each tone between age and left and right TP (upper row; 4- to 16-Hz activity averaged from 150 to 400 ms; age on *x*-axis and TP on *y*-axis). Bottom row scatterplots show associations for each tone between age and left and right ITC (upper row; 4- to 16-Hz activity averaged from 200 to 300 ms; age on *x*-axis and ITC on *y*-axis). Associations are shown for TDC (light gray) and ASD (black). (TIF 153 kb) Additional file 6:
**Online supplement.** (DOC 31 kb)

## Abbreviations

ASD: autism spectrum disorder
EEG: electroencephalography
EOG: electrooculogram
fMRI: functional magnetic resonance imaging
FWHM: full-width at half-max
GOF: goodness-of-fit
HPI: head position indicator
ITC: inter-trial coherence
M100: the ~100-ms component of the auditory evoked neuromagnetic field
M200: the ~200-ms component of the auditory evoked neuromagnetic field
M50: the ~50-ms component of the auditory evoked neuromagnetic field
MEG: magnetoencephalography
MRI: magnetic resonance imaging
PPI: probe position identification
ROI: region of interest
sMRI: structural magnetic resonance imaging
SRS: Social Responsiveness Scale scores
STG: superior temporal gyrus
TDC: typically developing children
TP: total power
