# Supplementary figures and images for: Auditory encoding abnormalities in children with autism spectrum disorder suggest delayed development of auditory cortex
Source: Mol Autism. 2015 Dec 30;6:69. doi: 10.1186/s13229-015-0065-5 (PMC4696177; doi:10.1186/s13229-015-0065-5)

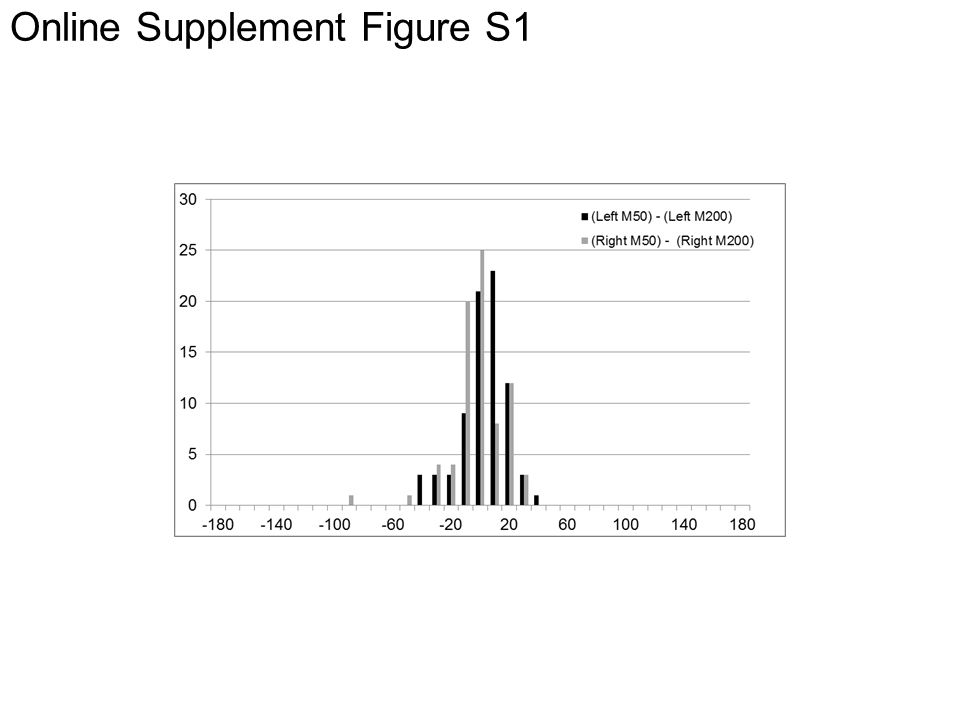

Supplement: Additional file 1: Figure S1. — For both the M50 and M200, the azimuthal dipole angle $\left(\theta =\mathrm{t}\mathrm{a}{\mathrm{n}}^{-1}\left(\frac{z\kern0.5em \mathrm{component}}{y\kern0.5em \mathrm{component}}\right)\right)$θ=tan−1zcomponentycomponent was examined as this best captures the variability in the tangentially oriented STG M50 and M200 dipole orientations. For each participant, the difference in the M50 and M200 dipole orientation was computed for the left and right hemisphere. The histogram shows very similar left and right M50 and M200 dipole orientations for almost all participants. (TIF 49 kb) [file 13229_2015_65_MOESM1_ESM.tif]

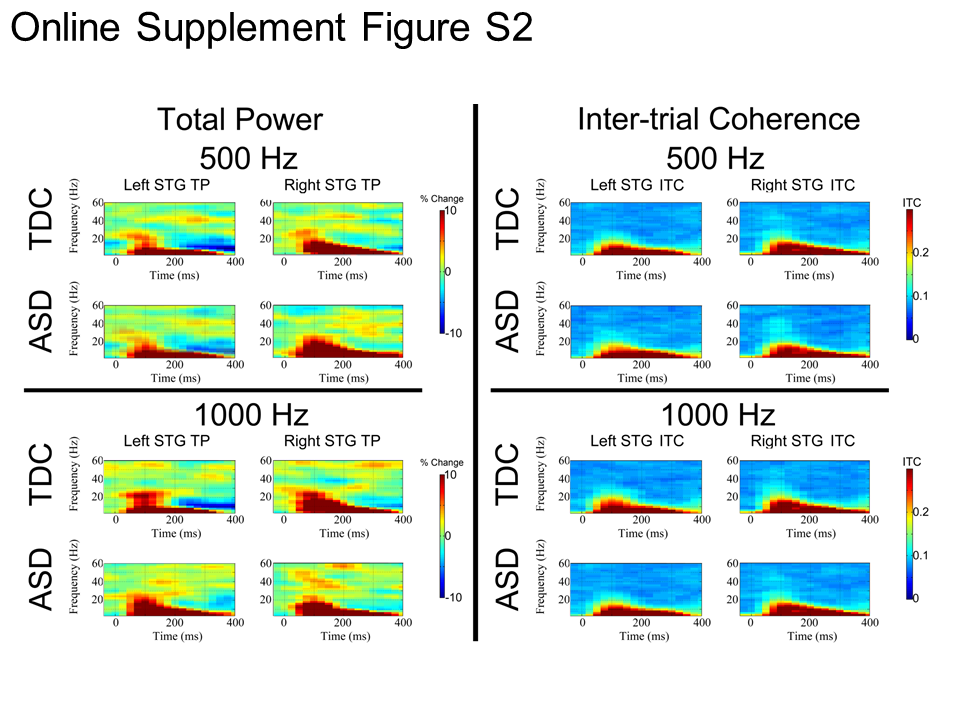

Supplement: Additional file 2: Figure S2. — The left panel shows grand average TP plots and the right panel ITC plots for each tone and each group. (TIF 338 kb) [file 13229_2015_65_MOESM2_ESM.tif]

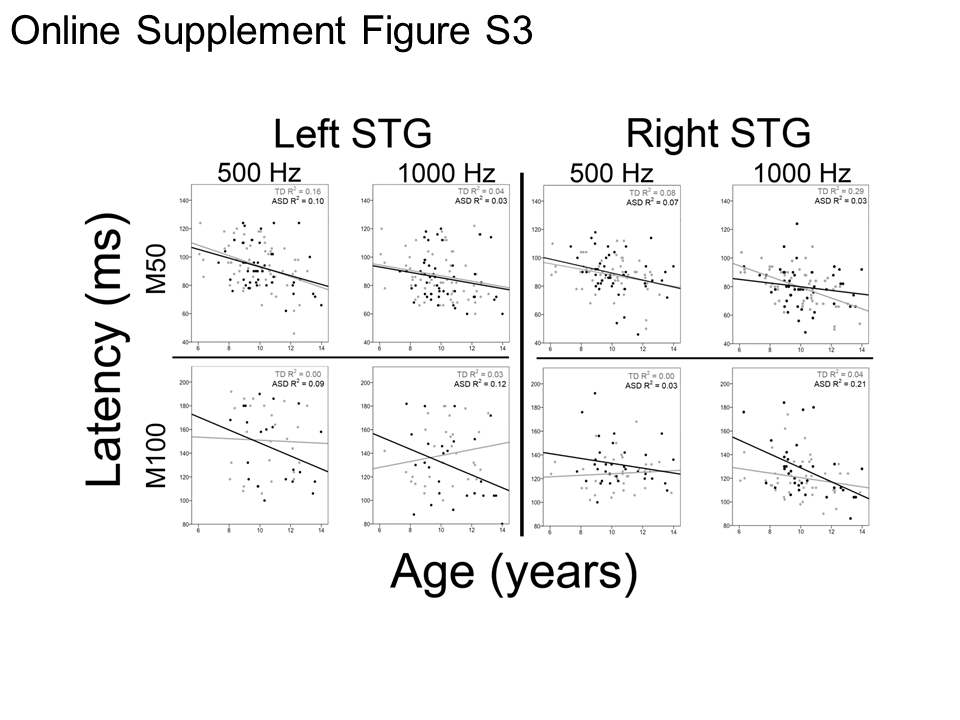

Supplement: Additional file 3: Figure S3. — Scatterplots showing associations for each tone between age and left and right M50 latency (upper row) and M100 latency (center row). Associations are shown for TDC (light gray) and ASD (black). The x-axis shows age and the y-axis latency. (TIF 128 kb) [file 13229_2015_65_MOESM3_ESM.tif]

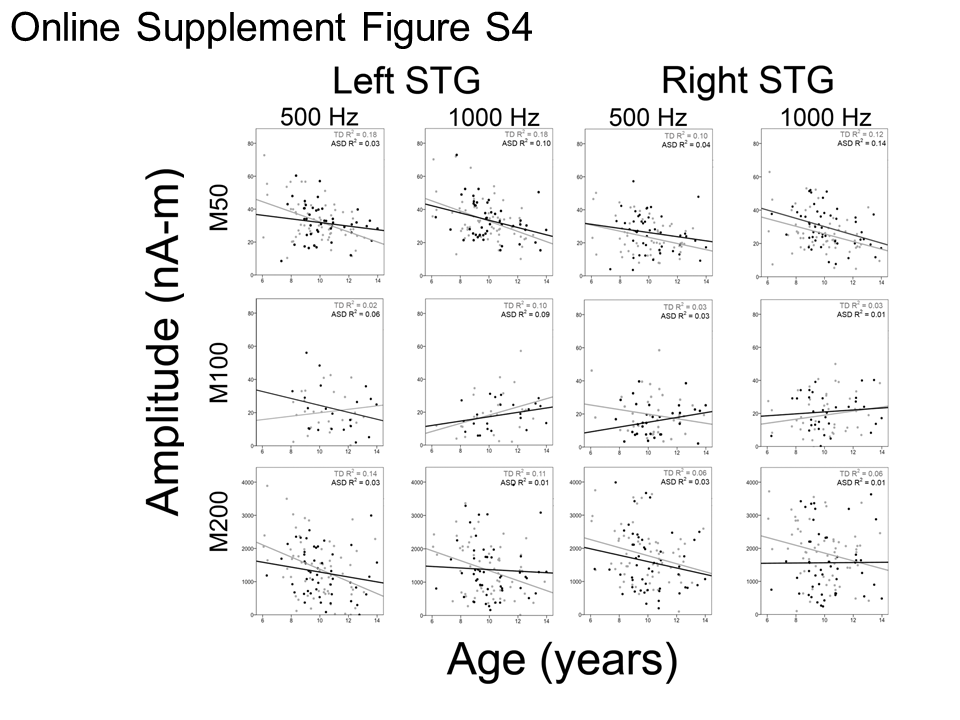

Supplement: Additional file 4: Figure S4. — Scatterplots showing associations for each tone between age and left and right M50 amplitude (upper row), M100 amplitude (center row), and M200 amplitude (bottom row). Associations are shown for TDC (light gray) and ASD (black). The x-axis shows age and the y-axis amplitude. For M200, the source strength measure is integrated over the full-width at half-max. (TIF 150 kb) [file 13229_2015_65_MOESM4_ESM.tif]

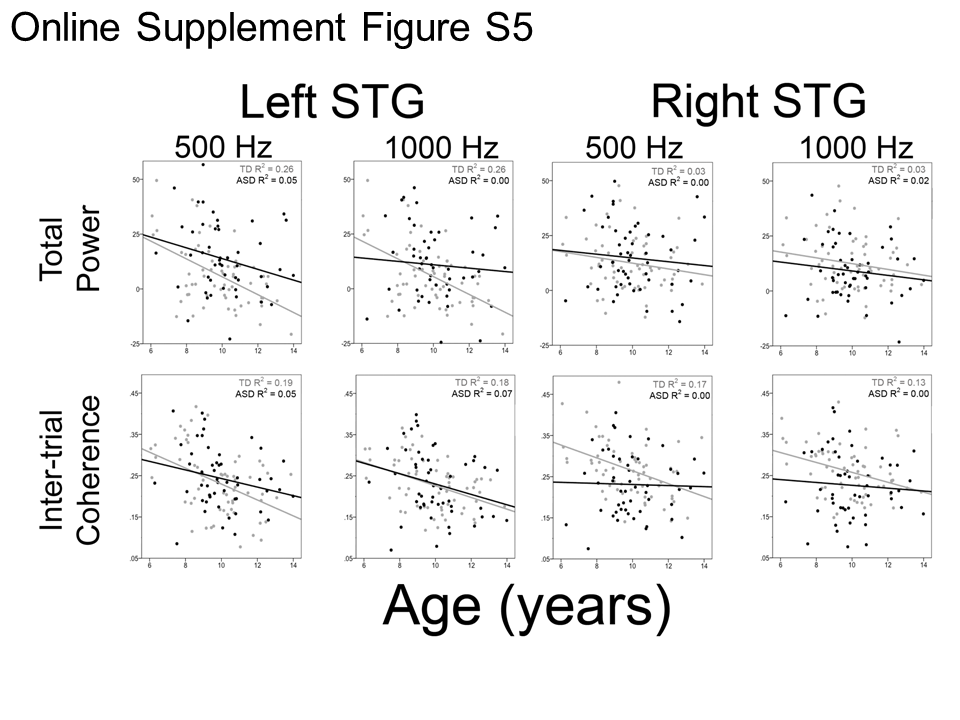

Supplement: Additional file 5: Figure S5. — Upper row scatterplots show associations for each tone between age and left and right TP (upper row; 4- to 16-Hz activity averaged from 150 to 400 ms; age on x-axis and TP on y-axis). Bottom row scatterplots show associations for each tone between age and left and right ITC (upper row; 4- to 16-Hz activity averaged from 200 to 300 ms; age on x-axis and ITC on y-axis). Associations are shown for TDC (light gray) and ASD (black). (TIF 153 kb) [file 13229_2015_65_MOESM5_ESM.tif]
