# Supplementary material for: Auditory encoding abnormalities in children with autism spectrum disorder suggest delayed development of auditory cortex
Source: Mol Autism. 2015 Dec 30;6:69. doi: 10.1186/s13229-015-0065-5 (PMC4696177; doi:10.1186/s13229-015-0065-5)
Supplement: Additional file 6: — Online supplement. (DOC 31 kb) [file 13229_2015_65_MOESM6_ESM.doc]

**Online Supplement:**

*M50 and M200 dipole orientations*

Online supplement Figure S1 histogram shows the difference in the M50 and M200 dipole orientation axes for the left and right hemisphere. The histogram shows very similar M50 and M200 dipole orientations (although opposed directions) for almost all subjects (i.e., normal distribution with large peak centered around 0°), indicating that the M50 and M200 time-frequency findings would be similar.

*Time-Frequency Findings: Grand average TP and ITC images*

Online supplement Figure S2 left panels show grand average M200 TP maps for TDC and ASD for 500 Hz (top left panel) and 1000 Hz (bottom left panel). Figure S2 right panels show ITC grand average maps for TDC and ASD for 500 Hz (top right panel) and 1000 Hz (bottom right panel).

*Time-Domain Findings: M50 and M100 latency, amplitude and age associations*

For each hemisphere, to examine how M50, M100, and M200 latency and amplitude are associated with age, hierarchical regressions were run with age entered first, group second, and the age x group interaction last, with M50, M100, and M200 latency or amplitude as the dependent variable.

**Latency:**

Online supplement Figure S3 scatterplots show age and M50 (upper row) and M100 (center row) latency associations for each group.

*M50:* Age and M50 latency associations were observed for both tones in both hemispheres in both groups: left 500 Hz (r = 0.34, slope = -3.3ms/year, *p* < 0.001), left 1000 Hz (r = 0.20, slope = -1.9ms/year, *p* < 0.05), right 500 Hz (r = 0.34, slope = -2.8ms/year, *p* < 0.01), and right 1000 Hz (r = 0.36, slope = -2.7ms/year, *p* < 0.001). None of the group main effects or the group interaction were significant.

*M100:* Age and M100 latency associations were not observed for left 500 Hz (r = 0.18, slope = -3.3ms/year, *p* > 0.05) or left 1000 Hz (r = 0.16, slope = -2.8ms/year, *p* > 0.05). For right 500 Hz, a significant main effect of group indicated later latencies in ASD (132 ms) than TDC (124 ms; *p* = 0.05). For right 1000 Hz, a main effect of age indicated similar age and M100 latency associations in TDC and ASD (r = 0.31, slope = -3.5ms/year, *p* < 0.01), and a marginally significant main effect of group indicated later latencies in ASD (128 ms) than TDC (120 ms).

**Amplitude:**

Online supplement Figure S4 scatterplots show age and M50 (upper row), M100 (center row), and M200 (lower row) amplitude associations for each group.

*M50:* Age and M50 amplitude associations were observed for both tones in both hemispheres in both groups: left 500 Hz (r = 0.32, *p* < 0.001), left 1000 Hz (r = 0.38, *p* < 0.05), right 500 Hz (r = 0.23, *p* < 0.05), and right 1000 Hz (r = 0.34, *p* < 0.001). For right M50 1000 Hz tones, a significant main effect of group indicated larger M50 responses in ASD versus TDC (*p* < 0.05).

*M100:* For left and right 500 Hz there were no significant main effects or interactions. Age and M100 amplitude associations were observed for left 1000 Hz (r = 0.30, p < 0.05). For right M100 1000 Hz, there were no significant main effects or interaction.

*M200:* For left 500 Hz, a main effect of age, F(1,111) = 5.16, *p* <0.05, indicated less M200 FWHM power in older participants. For left 1000 Hz FWHM power, a group X age interaction, F(1,105) = 3.90, *p < 0.05*, indicated age and FWHM power associations only in TDC (weaker M200 responses in older participants). For right 500 Hz FWHM power, a main effect of age, F(1,112) = 4.32, *p* <0.05, indicated weaker M200 responses in older participants. For right 1000 Hz FWHM power, none of the main effects or interaction were significant.

*Time-Frequency Findings: age associations*

Online supplement Figure S5 scatterplots show age and TP (upper row) and ITC (lower row) associations for each hemisphere, tone, and group. For left 500 Hz TP, a main effect of age, F(1,112) = 13.75, *p* < 0.001, indicated an association between age and TP (r = 0.33), and a main effect of group, F(1,111) = 7.89, *p* < 0.01, indicated greater TP in ASD versus TDC. For right 500 Hz TP, there were no main effects or significant interaction. For left 1000 Hz TP, a marginally significant main effect of group, F(1,111) = 3.60, *p* = 0.06, indicated greater TP in ASD versus TDC, and simple effect analyses of a left 1000 Hz age x group interaction, F(1,110) = 4.21, *p* < 0.05, indicated TP and age associations in TDC (r = 0.51) but not ASD (r = 0.08). A trending right 1000 Hz main effect of age, F(1,111) = 2.99, *p* = 0.09, indicated an association between age and TP in TDC and ASD (r = 0.16).

For left 500 Hz ITC, a main effect of age, F(1,112) = 15.52, *p* =< 0.001, indicated an association between age and ITC in TDC and ASD (r = 0.35). For right 500 Hz ITC, a main effect of group, F(1,111) = 6.72, *p* < 0.05, indicated greater ITC in TDC (0.27) versus ASD (0.23), and simple effect analysis of a marginally significant age X group interaction, F(1,110) = 3.45, *p* = 0.07, indicated an association between age and ITC in TDC (r = 0.42, p < 0.001) but not ASD (r = 0.03, p > 0.05). For left 1000 Hz ITC, a main effect of age, F(1,112) = 13.91, *p* < 0.001, indicated an association between age and ITC (r = 0.33). For right 1000 Hz ITC, a main effect of age, F(1,112) = 5.51, *p* < 0.05, indicated an association between age and ITC (r = 0.22), and a main effect of group, F(1,111) = 6.07, *p* < 0.05, indicated greater ITC in TDC versus ASD.

Although M200 TP and ITC regression analyses provided support for maturational delays in ASD, the evidence for this was not consistent. For example, group X age interactions were significant for left 1000 Hz TP but not left 500 Hz TP. Similarly, group X age interactions were significant for right 500 Hz ITC but not right 1000 Hz ITC. Stronger support for left TP and right ITC maturational abnormalities would have been obtained if weak age and TP and ITC associations in ASD were observed for 500 and 100 Hz tones. Examination of online supplement Figure 5, however, indicates that a failure to observe this more convincing pattern was due to insufficient power. Indeed, power analyses indicate that given the observed TDC and ASD correlations, and given a sample of 55 per group, the present study was very slightly underpowered to detect the left TP and right ITC interactions of interest: insignificant left 500 Hz TP interaction (TDC r = 0.51, ASD r = 0.22, power = ~70%) versus significant left 1000 Hz interaction (TDC r = 0.51, ASD r = 0.00, power = ~98%); significant right 500 Hz ITC interaction (TDC r = 0.41, ASD r = 0.00, power = ~87%) versus insignificant left STG 1000 Hz (TDC r = 0.36, ASD r = 0.00, power = ~76%).
